# Geometric Morphometrics of Nine Field Isolates of *Aedes aegypti* with Different Resistance Levels to Lambda-Cyhalothrin and Relative Fitness of One Artificially Selected for Resistance

**DOI:** 10.1371/journal.pone.0096379

**Published:** 2014-05-06

**Authors:** Nicolás Jaramillo-O., Idalyd Fonseca-González, Duverney Chaverra-Rodríguez

**Affiliations:** Instituto de Biología, Facultad de Ciencias Exactas y Naturales, Universidad de Antioquia UdeA, Medellín, Colombia; New Mexico State University, United States of America

## Abstract

*Aedes aegypti*, a mosquito closely associated with humans, is the principal vector of dengue virus which currently infects about 400 million people worldwide. Because there is no way to prevent infection, public health policies focus on vector control; but insecticide-resistance threatens them. However, most insecticide-resistant mosquito populations exhibit fitness costs in absence of insecticides, although these costs vary. Research on components of fitness that vary with insecticide-resistance can help to develop policies for effective integrated management and control. We investigated the relationships in wing size, wing shape, and natural resistance levels to lambda-cyhalothrin of nine field isolates. Also we chose one of these isolates to select in lab for resistance to the insecticide. The main life-traits parameters were assessed to investigate the possible fitness cost and its association with wing size and shape. We found that wing shape, more than wing size, was strongly correlated with resistance levels to lambda-cyhalothrin in field isolates, but founder effects of culture in the laboratory seem to change wing shape (and also wing size) more easily than artificial selection for resistance to that insecticide. Moreover, significant fitness costs were observed in response to insecticide-resistance as proved by the diminished fecundity and survival of females in the selected line and the reversion to susceptibility in 20 generations of the non-selected line. As a practical consequence, we think, mosquito control programs could benefit from this knowledge in implementing efficient strategies to prevent the evolution of resistance. In particular, the knowledge of reversion to susceptibility is important because it can help in planning better strategies of insecticide use to keep useful the few insecticide-molecules currently available.

## Introduction

*Aedes aegypti* (Linnaeus) is an urban mosquito which transmits several viruses, mainly the four serotypes of dengue virus, of increasing concern to public health policies. The global burden of dengue is huge; Bhatt et al. [1] estimates there are 390 million dengue infections per year, with only 96 million of which are apparent.

Currently, there are no available licensed vaccines or specific drugs to prevent infection, so the more effective way to reduce transmission is vector control or interruption of vector-human contact [2]. However, so far all efforts to control *Ae. aegypti* are threatened by the rapid and widespread emergence of insecticide-resistance [2], [3]. Insecticide resistance is a heritable trait and thus subject to natural selection [4]. In the absence of insecticides, mosquitoes carrying resistant alleles frequently exhibit a diminished fitness relative to susceptible ones; but that fitness costs vary between species and even between populations of the same species in time and space [5], [6]. Brown et al. [7] demonstrated that this natural variation of fitness costs can qualitatively affect the economic prescriptions of optimal control models of mosquitoes. Seizing on fitness costs, an effective integrated management of insecticide-resistance requires monitoring resistance and understanding the underlying evolutionary biology of mosquitoes [2], [7].

It is therefore essential to perform both periodic surveillance of insecticide-resistance and research on the factors that promote or prevent the emergence of resistance. Research on the variation of components of fitness can help to develop policies for effective integrated management and control, identifying when and how agencies must make changes in the types of insecticides to keep susceptible vector populations and preserve the utility of insecticides molecules [3], [6], [7].

Size and shape could be strongly associated with fitness [8], [9]. Geometric morphometrics is a powerful analytical tool for studying size and shape variability [10]. In turn, life tables provide the basis for studying the integrated life history characteristics that we would expect to be connected with fitness [11]. Then, combining geometric morphometrics with construction of life tables might improve our understanding of the trade-offs behind the outcomes of relative fitness.

In this work, we investigated the variation in wing size and shape of nine *Ae. aegypti* field isolates, which showed different resistance ratios 50 (RR_50_) to lambda-cyhalothrin, a widely used adulticide pyrethroid. To deepen the relationship between morphology, insecticide resistance, and fitness, we chose one of those isolates for laboratory selection of resistance because it exhibited the highest natural resistance ratio against lambda-cyhalothrin. Relative fitness cost and morphometric variation of the selected line was measured and compared with a non-selected line from the same isolate and with the susceptible reference Rockefeller strain (ROCK). The selected and the non-selected lines shared the same genetic background, which solves one of the most important weaknesses of many works: comparisons of unrelated resistant and susceptible strains. Resistant and susceptible strains may differ in many other genes than those involved in resistance, which is particularly relevant because populations from different geographical origins often differ in life histories [12].

## Materials and Methods

### Insects

The first level of comparison included nine field isolates. These were from two very distant Colombian municipalities, Cúcuta and Quibdó, located at the northeast and at the west of the country, respectively (Table 1, Fig. S1). We used the ROCK strain as reference of susceptibility to lambda-cyhalothrin [13]. The different levels of natural resistance were calculated as RR_50_ for all isolates, a procedure that we explained in detail here under the section: *Bioassays*.

**Table 1:** Origin and resistance status to lambda-cyhalothrin of *Ae. aegypti* isolates.

MUNIC.	NEIGHB.	COORD. (north, west)	RR_50_-LAMB	DATE OF COLLEC.	CODE	N
Cúcuta	Belén	7°52′18.42", 72°32'4.02"	8.08	06/28/08	BEL	52
Cúcuta	Comuneros	7°54′54.42", 72°31′44.82"	24.23	07/09/07	COM	51
Cúcuta	El Contento	7°53′9.66'', 72°30′43.56''	8.75	04/04/08	CONT	54
Cúcuta	Guaimaral	7°54′56.7'', 72°29′48.96''	23.00	08/14/08	GUAI	51
Cúcuta	La Libertad	7°53′23.88'', 72°28′49.38''	17.50	12/21/07	LIBE	50
Quibdó	Cristo Rey	5°41′39'', 76°39′31"	9.50	04/26/08	CREY	50
Quibdó	Jardín	5°41′01'', 76°38′42"	8.00	04/26/08	JARD	52
Quibdó	Playita	5°40′44'', 76°39′17"	10.25	04/26/08	PLAY	50
Quibdó	Porvenir	5°41′49'', 76°38′58''	1.60	04/26/08	PORV	51
Reference	The Rockefeller strain		1.00		ROCK	49
TOTAL						510

MUNIC.: Municipality, NEIGHB.: Neighborhood, COORD.: Geographical coordinates, RR_50_-LAMB: Resistance Ratios 50 to lambda-cyhalothrin, DATE OF COLLEC.: date of collection. N: sample size; CODE: isolate code; Ref.: Reference of susceptibility.

The second level of comparison included both the non-selected and selected lines for resistance to lambda-cyhalothrin from Comuneros (COM) isolate, which were followed in laboratory for 20 generations.

### Ethics Statement

Neither humans nor endangered or protected animals were involved in this study. The procedure of feeding mosquitoes on mice was adjusted to the guidelines established by the Ethics Committee for Animal Experiments of the University of Antioquia, which approved the study as stated in Act 30 of the April 6, 2006.

### Mosquito rearing

Larvae and pupae from each isolate were collected from different types of natural and man-made container habitats, using standardized protocols to determine aedic indexes. Within each municipality, isolates were separated by 1.5 to 5 km, and within each isolate we sampled at random 15–20 breeding-sites, thus avoiding the collection of larvae and pupae descendants from the same female. Samplings were coordinated with the “Instituto Departamental de Salud, Dirección de Vigilancia en Salud Pública” from municipality of Cúcuta, and with the “Departamento Administrativo de Salud, Unidad de Control de Vectores” from municipality of Quibdó, which helped with trained field-assistants and with transport.

The collected larvae and pupae were taken to the laboratory using containers filled with water from their habitats and afterward they were transferred to plastic pans of 2 L filled with dechlorinated water. Larvae were fed with fish food and the emerged adults were transferred to cages (25×25×25 cm) where plastic cups covered internally with paper towels and filled with water were placed for oviposition. Mosquitoes were fed *ad libitum* with a 10% (w/v) sugar solution and, additionally, females were fed each two days on mice Balb/c 3–4 months old. Insectary colonies were raised to adults at 28±2°C, 70±5% RH and a photoperiod of 12 hours light/dark.

### Biossays

The resistance ratios 50 (RR_50_) were evaluated in larvae from nine field isolates and each generation for the selected and the non-selected lines of mosquitoes from the parental COM population and the ROCK strain using the WHO protocols [14], [15]. Groups of 20 fourth instar larvae were exposed for 24 h at least to five different concentrations of lambda-cyhalothrin (purchased from Chem Service Inc., West Chester, PA, USA) producing mortalities between 2 and 90%. The insecticide was prepared from a 1 mL stock solution (10 ug dissolved in 1 ml of acetone) in 99 mL of dechlorinated water; the control sample contained a stock solution of acetone instead of insecticide. Each insecticide concentration was tested in triplicate and each bioassay was performed twice for each field isolate and generation.

Larval mortality data obtained after 24 h of exposure to each insecticide-concentration and that of the controls were used to calculate lethal concentration 50 (LC_50_) through Log-Probit linear regression [16]. Mortality data were corrected by the Abbot's formula [17]. The RR_50_ was calculated for each generation by dividing the LC_50_ of the field isolate and both the selected and the non-selected lines by the LC_50_ of the ROCK strain. The RR_50_ values were interpreted according to Mazzarri and Georghiou [18] thus: a value <5-fold is taken as low or “susceptible”; a value between 5-fold and10-fold is taken as moderate or “tolerant”, and a value >10-fold is taken as high or “resistant”.

The resistance status was confirmed in adults using the CDC bottle bioassays [19]. Each field isolate and generation of the selected and non-selected lines from COM isolate and the ROCK strain were evaluated relative to diagnostic doses (DD) and times previously known to kill 100% of mosquitoes from the ROCK strain: 6.25 ug/15 min [20]. For this, we exposed to lambda-cyhalothrin groups of 20 three days old females, fed only with a 10% (w/v) sugar solution, into 250 mL glass bottles internally coated with the insecticide. Each bioassay, tested in triplicate, consisted of four treated bottles (impregnated with DD of the insecticide) and one control bottle (impregnated only with acetone). Mortality was checked each 5 min for 2 h. The mortality criteria included mosquitoes with difficulty in flying or as resting on the bottle's surface.

### Selection of resistance to lambda-cyhalothrin

The COM isolate from the municipality of Cúcuta was chosen as the parental for selection of insecticide-resistance because it showed the highest natural RR_50_ to lambda-cyhalothrin (24.23X) (Table 1). The first offspring (F1) was randomly separated into two batches, one to be exposed to lambda-cyhalothrin, each generation; the other batch was kept in parallel but without exposure to insecticide. From 1000 to 2000 larvae (divided in groups of 250) were exposed during 24 h to the LC_50_ of lambda-cyhalothrin estimated by dose-response assays. The emerged adults from survivor larvae were reared at the same laboratory at the above mentioned conditions. This procedure was iterated for 20 generations. The LC_50_ increased in each generation as the larval and adult bioassays showed a progressive increase in the insecticide-resistance.

The same procedure was performed for the other batch of 1000 to 2000 larvae from COM isolate and for the ROCK strain; but using a solution of acetone and water in the same proportion of LC_50_ instead of lambda-cyhalothrin.

We took care to keep an equal proportion of parental males and females (500 to 700 mosquitoes) for each generation in all the treatments.

### Geometrics morphometrics

**Figure 1:**
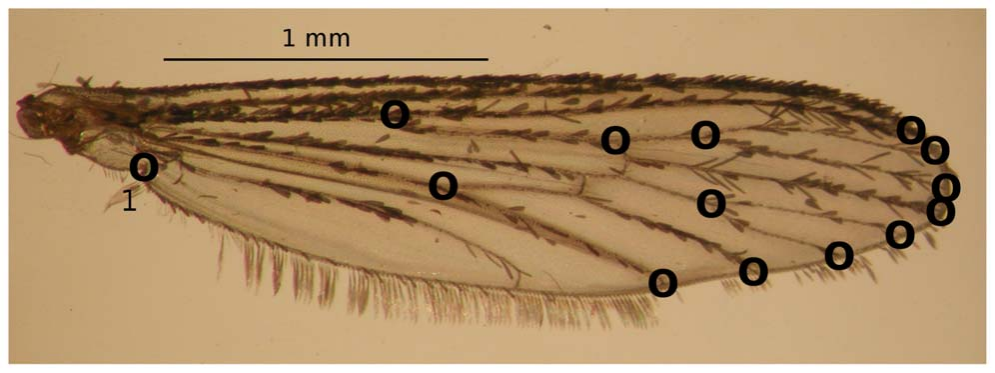
Arrangement of landmarks digitized on right and left wings of 758 *Aedes aegypti* females. The landmarks were digitized in a clockwise sequence from the number one.

We studied a total of 758 females chosen at random from each field isolate and the ROCK strain (n = 510), and the selected and non-selected lines at F9 and F20 generations (n = 248). The following procedures were performed for the isolates from field, the selected and non-selected lines and the ROCK strain. Right and left wings were carefully dissected and mounted between microscope slides and cover slips, and afterward were photographed taking care to avoid optical distortion of the microscope's peripheral visual field. We selected 14 type I landmarks (sensu [21]) on vein intersections of hemelytra (Fig. 1). Landmarks were digitized to Cartesian coordinates and those corresponding to right and left wings were averaged to eliminate possible bias because of fluctuating asymmetry. The measurement error was estimated by the Pearson correlation coefficient in two set of coordinates taken on the same subsample of 30 specimens chosen at random from one of the isolates [22].

To analyze wing size variation, we used an isometric estimator, the centroid-size (CS), which was calculated as the square root of the sum of the squared distances between the center of the configuration of landmarks and each landmark [21]. The CS variation was analysed with the Kruskal-Wallis rank sum tests and the post hoc pairwise Wilcoxon rank sum tests, and their significance was tested with a permutation procedure (10,000 permutations), with p-values corrected with the Bonferroni method. The relationships in wing size were visualized through a violin plot [23] and a dendrogram built with the UPGMA method. The cophenetic correlation coefficient [24] was used to measure the correlation between the distances used to construct the UPGMA dendrogram and the distances implied by the dendrogram itself. To analyse the association between wing size and resistance, we performed a linear regression of CS on RR_50_.

To verify the correlation between wing centroid-size and body size, we performed a linear regression of the dry weight of a sample of 150 females on the CS. The sample of 150 females was chosen randomly from the ROCK strain and the selected and the non-selected lines at F20 generation. They were first dried at 37°C in the oven with silica gel bags for 48 h and then weighed in groups of five on an analytical balance (OHAUS Explorer mod. AV114, 110 g×0.1 mg).

To analyze wing shape variation, the raw coordinates matrix was transformed with the generalized Procrustes analysis [25]. The algorithm consists of a series of iterative superimpositions of homologous landmarks to adjust them by scale, translation and rotation. The iterations allowed computing the mean wing shape which becomes a reference to compute the distances between individuals. Then, the resulting variables are shape variables which can be analysed with multivariate statistical methods. We analysed shape variables by relative warps analysis which corresponds to a principal component analysis of wing shape variation [21]. The relationships in wing shape were visualized by a scatter plot of the individuals on the two first relative warps and by a dendrogram built by the UPGMA method. The dendrogram fidelity was verified by the cophenetic correlation coefficient [24]. Mahalanobis distances between isolates means were computed and their significance was tested with a permutation procedure (10,000 permutations). Furthermore, the Mahalanobis distances were used to perform a jack-knife cross-validated classification [26].

### Allometry

Allometry was tested using a multivariate regression of wing shape on wing size, and their significance was tested with a permutation procedure (10,000 permutations). MANCOVA was used to test for a common allometric slope. When a common slope was deemed appropriate, residuals of the regression of shape on centroid size were used for downstream analyses. The regression was scaled by size to test for differences in shape without the size effect.

### Life tables and fitness cost

Life history was evaluated for the following groups of insects: parental COM population, the ROCK strain, and both the selected and non-selected lines at F9 and F10 generations. The model that we studied for life histories assumed ideal conditions for laboratory with discrete, non-overlapping generations. For each group, five cohorts (five replicates) each of 100 larvae of 12-h old were kept at the same conditions mentioned above, but in plastic pans of 500 ml. Daily register of larval mortality and molting were recorded until pupation. Pupae were transferred to new pans with freshly dechlorinated water and female and male emergence was daily recorded. For each group, four cohorts (four replicates) each of 25 females and 25 males of 12 hours old were kept in boxes of 25×25×25 cm, and fed *ad libitum* with a 10% sugar solution (w/v) and daily on mice Balb/c aged 3–4 months old at the same environmental conditions previously mentioned. One 30 mL plastic cup per cage covered internally with paper towels and filled with water were disposed for oviposition. Daily mortality was recorded at 7:00 h and 19:00 h, until the last mosquito was dead. Oviposition paper towels were daily removed one time per day, and all the eggs were counted under the microscope. This experiment was repeated for the 200 females and 200 males used.

The vital parameters studied were: average time in days to develop to pupae, average time in days to develop to adults, proportion of male and female emergence, hatchability, age-specific survival of females (*l_x_*: number of females alive at beginning of stage x) and fecundity (*m_x_*: average number of eggs produced per female). Kruskal-Wallis rank sum tests or ANOVA were used for comparisons, and when significance was found, pair-wise comparisons were performed with Tukey tests corrected by Bonferroni method. Age-specific survival comparisons were performed by log-rank analysis and the Kaplan-Meier method was used to build survival curves.

These parameters were used to prepare life tables from which the following demographic growth estimates were calculated: generation time (
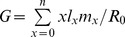
), where x is the proportion of individuals in day x), net reproductive rate per generation (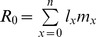), and intrinsic rate of increase in days (r = ln*R_0_*/*G*). Both, *R_0_* and *r* can be estimators of fitness, but here we used *r* to estimate the relative fitness because it combines information from both survivorship and fecundity, in terms of *R_0_* and *G*. The *r* value is the most comprehensive life table parameter and therefore the most useful to explain evolutionary changes in generation time [27]. We use the Euler-Lotka equation (
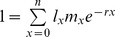
) as a more accurate way to calculate *r* [28] and thus the relative fitness. The *R_0_* and *r* demographic parameters were compared across groups by using ANOVA and post hoc pair-wise Tukey's studentized range tests.

### Software

Most of the statistical analyses were performed using the R language [29]. Geometric morphometrics and multivariate analyses of shape were performed using the CLIC package [26]. The tpsRelw program [30] was used to make deformations grids for the first and second relative warps on the mean configuration. The Probit program [31] was used for calculating LC_50_ through Log-Probit linear regression.

## Results

### Repeatability of landmarks

Size and shape showed fairly good precision in the digitalization of landmarks in two sets of repeated wings (0.998 for centroid-size, and 0.902, 0.868, 0.929 for the first three relative warps).

### Geometric morphometrics and resistance to lambda-cyhalothrin of field isolates

**Figure 2:**
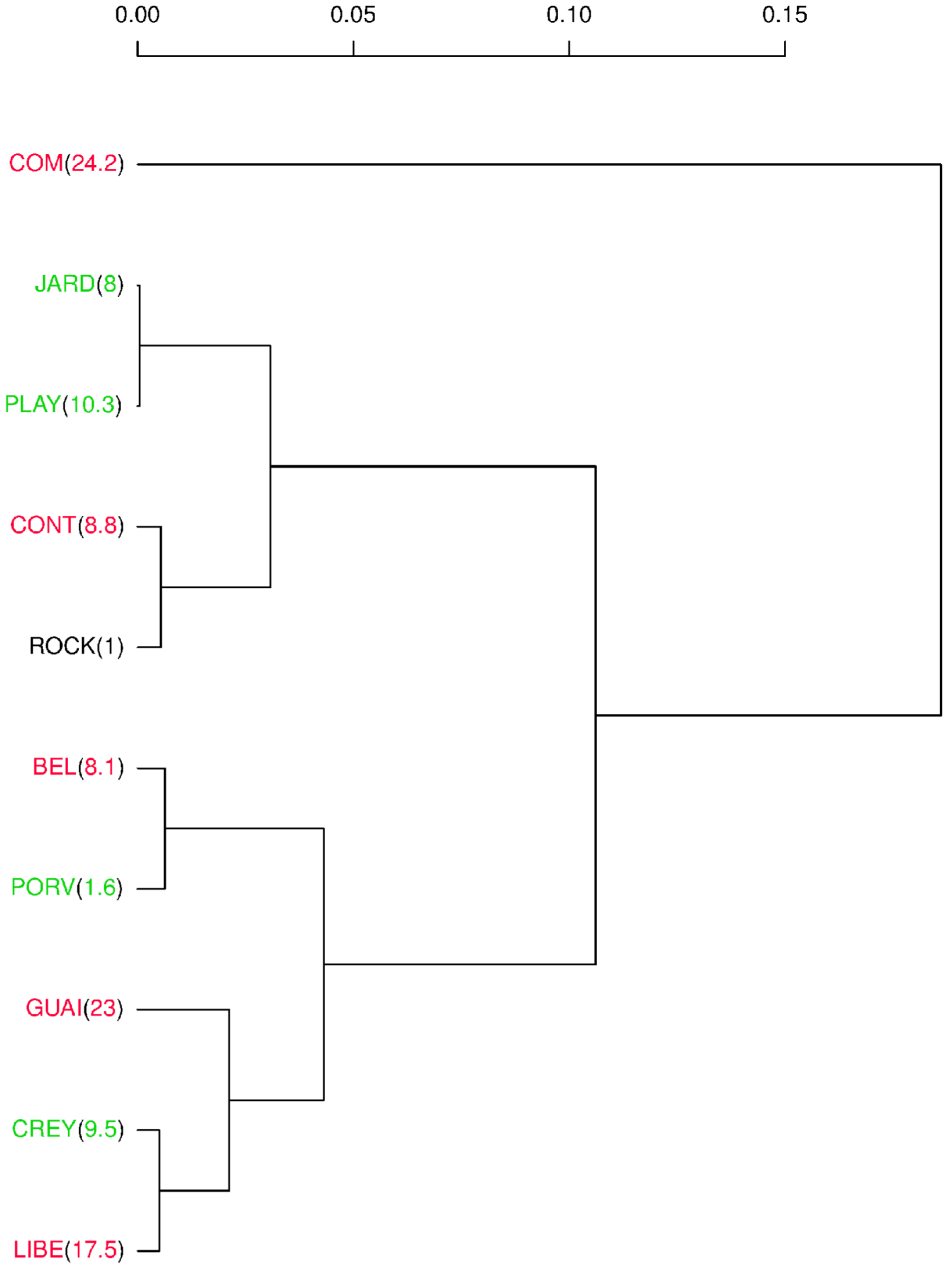
Dendrogram showing the relationships in wing size of isolates from Cúcuta and Quibdó. Between parentheses is the RR_50_ to lambda-cyhalothrin. Cophenetic correlation coefficient: 0.892522. isolates from Cúcuta are colored in red, and those from Quibdó in green ROCK: the Rockefeller strain.

**Figure 3:**
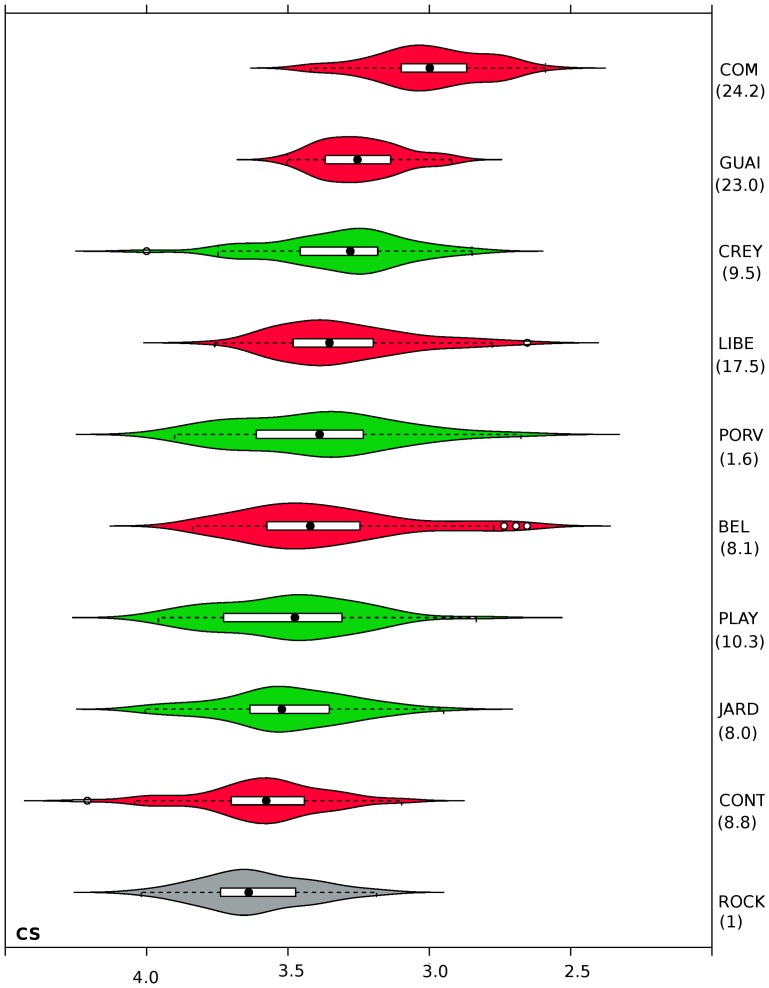
Wing size variation among *Aedes aegypti* isolates from field. Violin plots enclose box plots. Each box is divided by the median (black circles), which top and bottom correspond to 25th and 75th quartiles, respectively. Isolates from Cúcuta are colored in red, and those from Quibdó in green. Between parentheses is the RR_50_ to lambda-cyhalothrin. CS: centroid-size.

Kruskal-Wallis rank sum test showed significant differences in wing size among isolates from the cities of Cúcuta and Quibdó and the susceptible reference ROCK strain (χ2 = 187.4598, df = 9, *P* = 2.2×10^−16^). COM isolate displayed the highest resistance against lambda-cyhalothrin (Table 1) and was the most different in size (Fig. 2) and included the smallest individuals (Fig. 3). Additionally, most of the isolates that exhibited higher resistance included the smaller individuals. However, the ROCK strain contained the biggest individuals (Fig. 3), but the correlation between resistance and size was not perfect (Fig. 2). The ROCK strain (RR_50_ = 1) was not statistically different from the other three isolates (Wilcoxon rank sum test, *P*>0.3), which exhibited RR_50_ of 8.0, 8.8, and 10.3, but it was statistically different (Wilcoxon rank sum test, *P* = 0.005) from an isolate with a RR_50_ of 1.6 (Fig. 2). Linear regression of wing size (CS) on RR_50_ was significant, but the coefficient of determination was moderate (adjusted R^2^ = 0.2307, F = 153.6, df = 1 and 508, *P*<2.2×10^−16^). Furthermore, the dendrogram (Fig. 2) shows that similar wing sizes are found in isolates belonging to different municipalities far between (Cúcuta and Quibdó) or in isolates from different climates.

**Figure 4:**
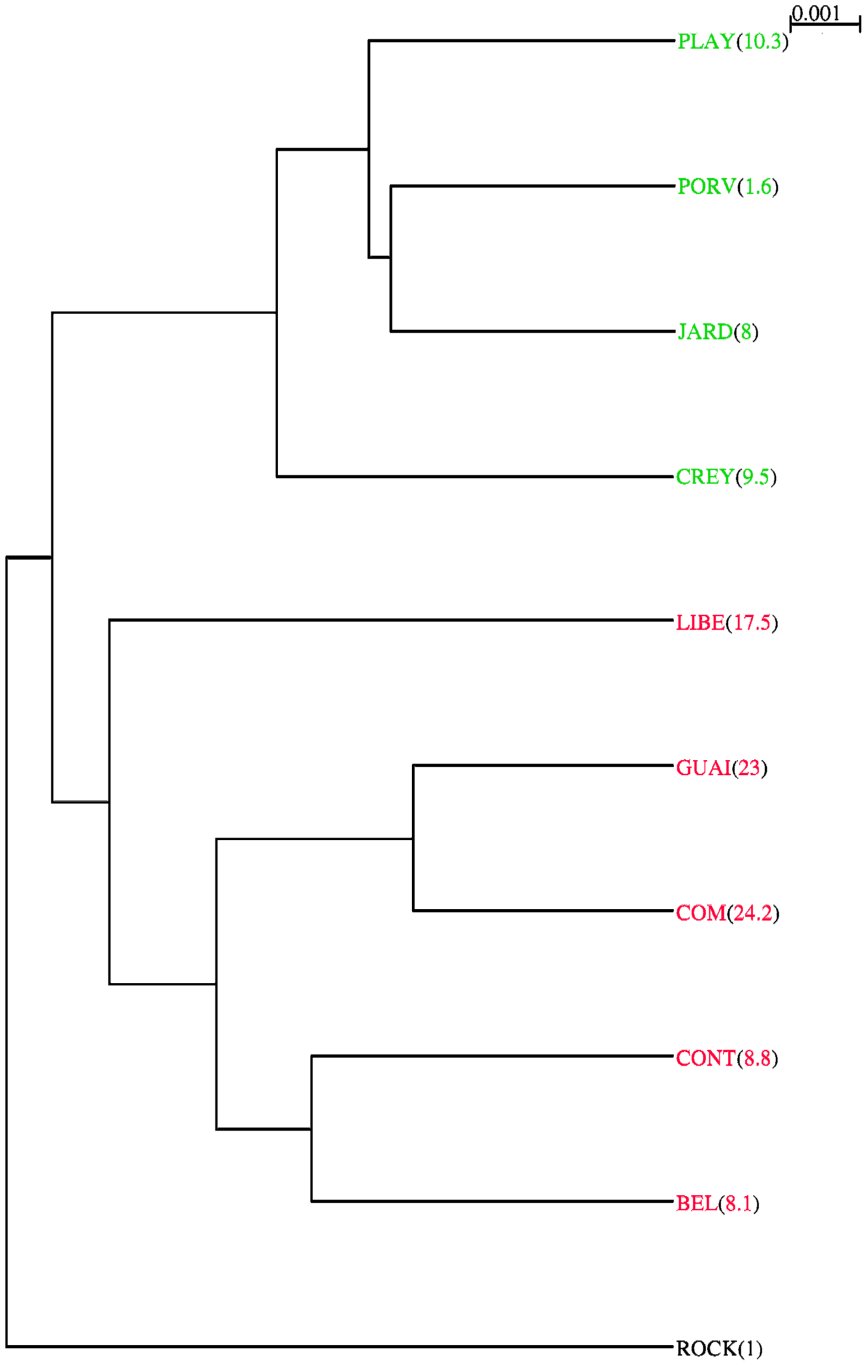
Dendrogram showing the relationships in wing shape of isolates from Cúcuta and Quibdó. Cophenetic correlation coefficient: 0.8935737. Isolates from Cúcuta are colored in red, and those from Quibdó in green. Between parentheses is the RR_50_ to lambda-cyhalothrin. ROCK: the Rockefeller strain.

Wing shape was also different among isolates. Outcomes of relative warp analysis visualized by a dendrogram showed two levels of similarity in wing shape; the first one coincides to geographical origin (three clusters corresponding to the cities of Cúcuta and Quibdó, and to the ROCK strain, respectively), and the second one to RR_50_ against lambda-cyhalothrin (Fig. 4). The dendrogram also shows a stronger relationship of wing shape with RR_50_, than that of wing size (Fig. 4). Mahalanobis distances were significantly different between the ROCK strain, and the cities of Cúcuta, and Quibdó (from 10,000 permutations, no Mahalanobis distance was equal or higher than observed: *P*<0.0001). Isolates within cities of Cúcuta and Quibdó were also different except for two of them (Table 2). Cross-validated classification of each individual to its original isolate shows fairly good correct classification rates of the three clusters (ROCK: 85%, Cúcuta: 75%, Quibdó: 77%) but moderate within each cluster (27% to 66%).

**Table 2:** Differences in wing shape among *Ae. aegypti* isolates.

	ROCK	BEL	COM	CONT	GUAI	LIBE	CREY	JARD	PLAY	PORV
ROCK	0	0.0000	0.0000	0.0000	0.0000	0.0000	0.0000	0.0000	0.0000	0.0000
BEL	3.5900	0	0.0000	0.0000	0.0000	0.0000	0.0000	0.0000	0.0000	0.0000
COM	4.7200	3.2600	0	0.0000	0.0000	0.0000	0.0000	0.0000	0.0000	0.0000
CONT	3.6000	2.7200	4.2500	0	0.0000	0.0000	0.0000	0.0000	0.0000	0.0000
GUAI	3.5300	2.0500	1.9800	2.8800	0	0.0000	0.0000	0.0000	0.0000	0.0000
LIBE	3.1700	2.7500	3.6900	3.0800	2.3500	0	0.0000	0.0000	0.0000	0.0000
CREY	3.8000	2.5300	3.4100	2.8000	2.4600	3.3400	0	0.0000	0.0000	**0.0615**
JARD	3.6600	2.3800	3.8600	2.9600	2.6700	3.2500	1.8000	0	0.0000	0.0000
PLAY	3.7500	2.8800	3.8800	3.2200	2.7800	3.5000	1.7500	1.7400	0	0.0002
PORV	3.3000	2.1300	3.3400	2.8300	2.3100	2.9800	1.3100	1.8300	1.7000	0

Mahalanobis distances are shown below diagonal and its corresponding *P* values are above diagonal. Using Bonferroni correction, a significant *P* value is <0.00111.

### Geometric morphometrics and resistance to lambda-cyhalothrin of artificially lambda-cyhalothrin selected and non-selected lines

The development of resistance was confirmed using bioassays on larvae and adults of COM isolate, on the selected and non-selected lines for lambda-cyhalothrin, and on the ROCK strain. Artificial selection increased the RR_50_ in each generation by a factor of 1.25X on average. The RR_50_ of larvae of parental COM was 24.23X, and the selected line at F9, F10, and F20 generations showed a RR_50_ of 225.4X, 91.52X, and 114.23X, respectively; However, the non-selected COM line showed for F9, F10, and F20 generations a RR_50_ of 10.0X, 12.13X, and 1.95X respectively. Adults followed in parallel way: parental COM was susceptible; but the selected line at F9, F10, and F20 generations showed a mortality of 25, 32, and 8%, respectively. However, the non-selected line at F9, F10, and F20 generations showed a mortality of 100, 100, and 97%, respectively (Table S1 and S2).

Wing centroid-size (CS) as estimator of global size was confirmed by a lineal regression of dry weight of body on CS (Adjusted R^2^ = 0.2625, F = 11.32, df = 1 and 28, *P* = 0.002236).

**Figure 5:**
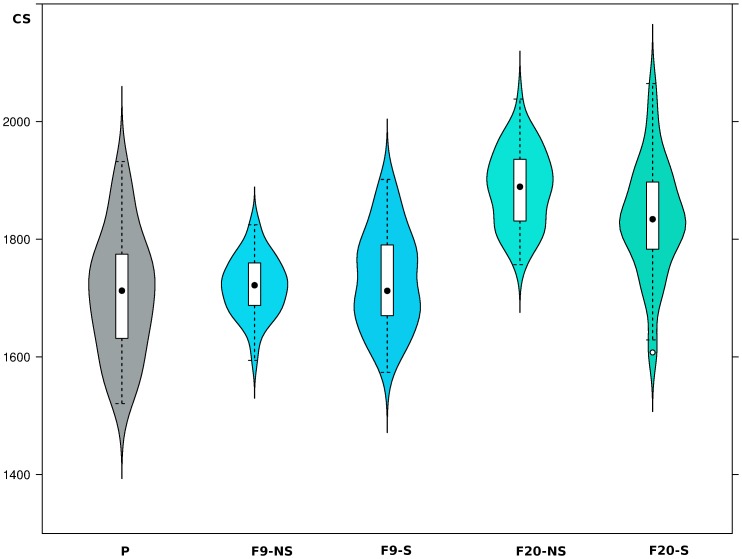
Wing size variation of both lambda-cyhalothrin selected and non-selected lines. Violin plots enclose box plots. Each box is divided by the median (black circles), which top and bottom correspond to 25th and 75th quartiles, respectively. CS: centroid-size; P: parental Comuneros isolate; F9-NS and F20-NS: ninth and twenty generations non-selected for lambda-cyhalothrin resistance; F9-S and F20-S: ninth and twenty generations selected for lambda-cyhalothrin resistance. Sample-sizes were 51 parental females, 42 F9-NS, 49 F9-S, 50 F20-NS, and 56 F20-S.

Kruskal-Wallis rank sum test showed differences in wing size of the selected and the non-selected lines, their parents, and the ROCK strain (χ2 = 111.1079, df = 4, *P*<2.2×10^−16^). Wing size did not show an appreciable change between the parental and the F9 generation of the selected and the non-selected lines (Fig. 5), but there was a significant increase in wing size of the F20 generation of both lines relative to its ascendants (Fig. 5). Notably, the selected and the non-selected lines at F9 generation did not show differences in wing size between them, but the non-selected F20 generation was bigger than the selected (Bonferroni corrected Wilcoxon *P* = 0.023).

**Figure 6:**
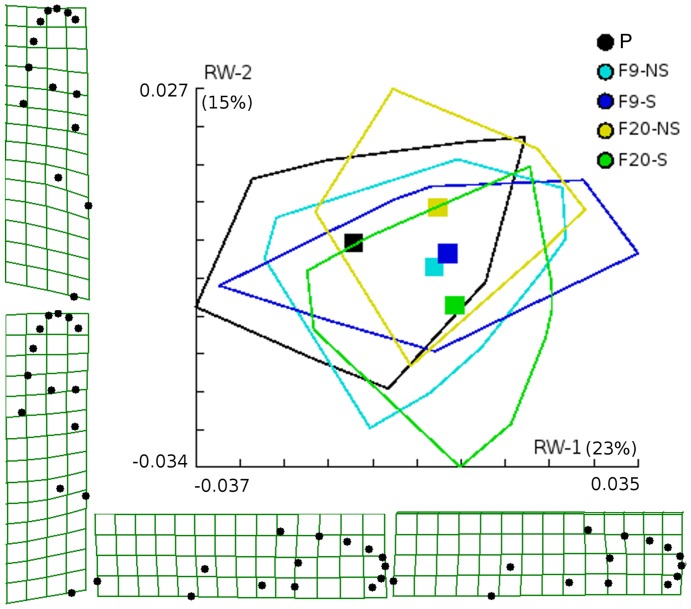
Scatterplot of the scores along the first two principal components (relative warps), the convex hull for each group and the average scores for each group. Convex hulls enclose individuals from parental and its descendants at ninth and twenty generations which were ordered on the two first relative warps. Squares represent the centroids (average scores for each group). To easy viewing of the group's centroids, the individual positions are not shown. Deformation grids for the extreme values of the first and the second relative warps on the mean configuration, are also shown. P: parental Comuneros isolate; F9-NS and F20-NS: ninth and twenty generations non-selected for lambda-cyhalothrin resistance; F9-S and F20-S: ninth and twenty generations selected for lambda-cyhalothrin resistance. Sample-sizes were 51 parental females, 42 F9-NS, 49 F9-S, 50 F20-NS, and 56 F20-S.

In similar way, wing shape becomes progressively different between the selected line and the non-selected one from the parental to the F9 and to the F20 generation (Fig. 6). Mahalanobis distances were statistically significant between the parental isolate and all of its descendants at F9 and F20 generations for both the selected and the non-selected lines and they were also significant between lines and generations (from 10,000 permutations, no Mahalanobis distance was equal or higher than observed, *P*<0.0001). Cross-validated classification of each individual to its original isolate shows fairly good correct classification rates of the three clusters, being the best classified the parental isolate, followed by the selected line at F20 generation, and after by the non-selected line of the same F20 generation (parental  = 82%, F20-S = 80%, F20-NS = 78%, F9-S = 67%, F9-NS = 59%).

### Allometry

For the field isolates, differences in wing shape were affected by wing size (multivariate linear regression of relative warps on CS after 10,000 permutations shows 0 values found equal or higher than those observed, *P*<0.0001); and the test for common slopes shows significance (Wilks lambda  = 0.548, F = 1.354, df = 216 and 3970.166, *P* = 0.0006), which means that a common allometric slope was not found. We repeated the MANCOVA, but excluding the ROCK strain, and the model shows significance as well (Wilks lambda  = 0.567, F = 1.295, df = 192 and 3215.4, *P* = 0.0048).

For the selected and non-selected lines, differences in wing shape were affected by the wing size (multivariate linear regression of relative warps on CS after 10,000 permutations shows 11 values found equal or higher than observed, *P* = 0.001); and the model of common slopes could be accepted (Wilks lambda  = 0.636, F = 1.079, df = 96 and 854.24, *P* = 0.294). Size-free analysis of wing shape confirmed statistic differences among parental isolate and its descendants of F9 and F20 generations of the selected and the non-selected lines but with higher differences at F20 generation than at F9 (after 10,000 permutations no Euclidian distances among lines and isolates were equal or higher than observed, *P*<0.0001).

### Life tables and fitness costs

The following parameters did not show statistical differences at P-level of 0.05 between the selected and the non-selected lines at F9 and F10 generations: average time to pupation, average time to eclosion, proportion of male and female emergence, and hatchability (Table S3).

**Figure 7:**
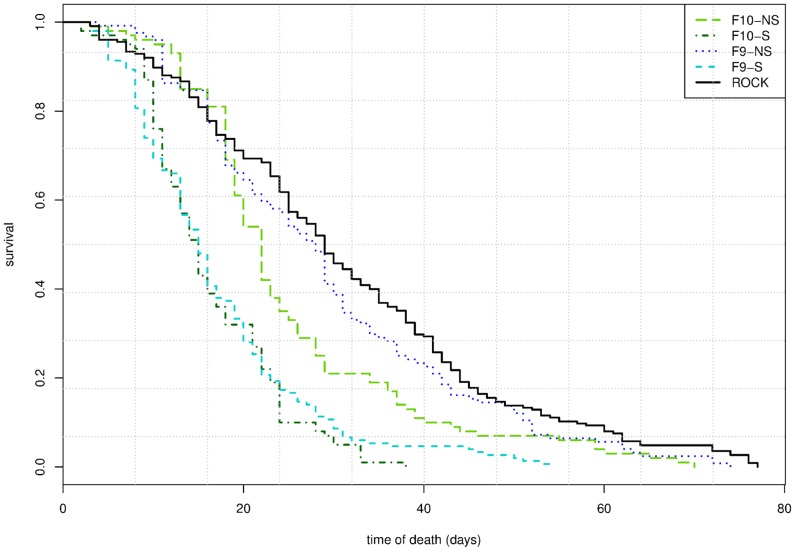
Survival analysis using Kaplan-Meier estimates. Curves represent daily survival of the Rockefeller strain (ROCK) females, and the ninth (F9-S) and tenth (F10-S) generations of the lambda-cyhalothrin selected line from COM isolate, and the ninth (F9-NS) and tenth (F10-NS) generations of the non-selected line from the same isolate. Sample-sizes were 100 females for both F10-S and F10-NS, 124 for F9-NS, 150 for F9-S, and 225 for the ROCK strain.

**Figure 8:**
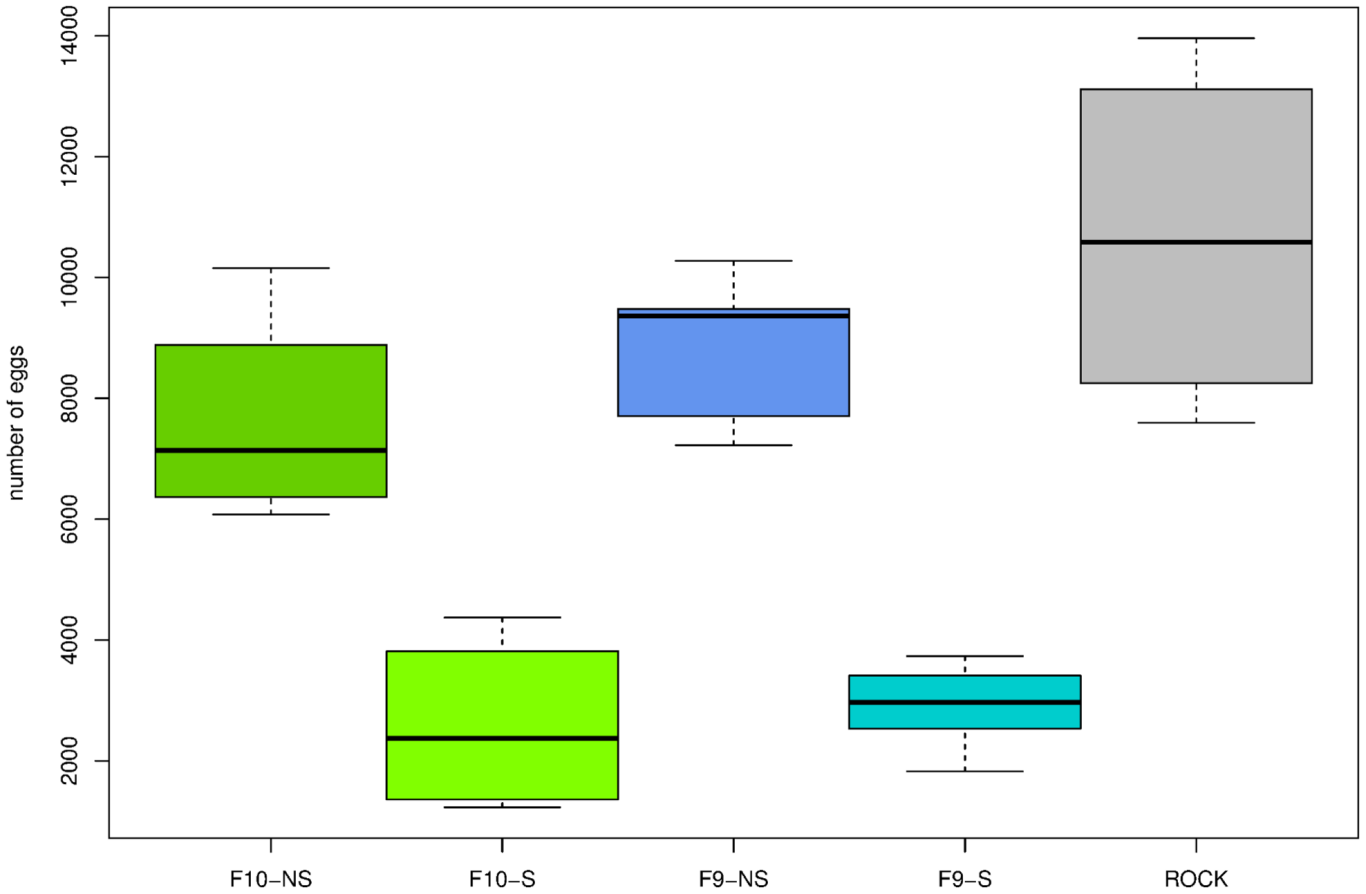
Box-plots showing the fecundity variation of both lambda-cyhalothrin selected and non-selected lines. Each box is divided by the median, which top and bottom correspond to 25th and 75th quartiles, respectively. ROCK: the Rockefeller strain, F9-S and F10-S: the selected line at ninth and tenth generations, and F9-NS and F10-NS: the non-selected line at ninth and tenth generations. Sample-sizes were 100 females for both F10-S and F10-NS, 124 for F9-NS, 150 for F9-S, and 225 for the ROCK strain.

However, the probability of survival was very different among tested groups (Log-Rank test: χ^2^ = 171, df = 4, *P*<0.0001; Fig. 7). The median survival of the selected line at F9 and F10 generations was 15 days for both of them; survival of the non-selected line was significantly higher (28 days for F9 and 22 for F10) as it was also for the ROCK strain (29 days). Fecundity was different among tested groups (ANOVA: F = 21.36, df = 4 and 15, *P* = 4.73×10^−6^). Pair-wise comparisons showed differences between the ROCK strain and the selected line at F9 and F10 generations (Tukey test  = 9.679, *P*  =  0.0001816, and Tukey test  = 9.68, *P*: 0.0001815, respectively (Fig. 8) but was similar between the ROCK strain and the non-selected line at F9 and F10 generations (Tukey test  = 1.575, *P*: 0.7971 and Tukey test  = 2.705, *P*: 0.3527, respectively).

Likewise, the net reproductive rate per generation (*R_0_*) and the intrinsic rate of increase in days (*r*) showed differences among the tested groups (ANOVA *R_0_*: F = 18.56, df = 4 and 15, *P* = 1.126×10^−5^; ANOVA r: F = 12.99, df = 4 and 15, *P* = 9.139×10^−5^). Both parameters were different between the ROCK strain and the selected line at F9 and F10 generations but were similar between the ROCK strain and the non-selected line at F9 and F10 generations (Table 3).

**Table 3:** Comparisons of the net reproductive rate per generation (*R_0_*) and the intrinsic rate of increase in days (*r*).

R_0_					
	ROCK	F9-NS	F9-S	F10-NS	F10-S
ROCK	0	0.9919	**0.0002812**	0.4515	**0.0002809**
F9-NS	1.6132	0	**0.0004547**	0.7027	**0.0004541**
F9-S	8.708	8.095	0	**0.003833**	1.00
F10-NS	2.433	1.82	6.275	0	**0.003825**
F10-S	8.71	8.096	0.001557	6.276	0

r					
ROCK	0	0.9991	**0.0008817**	0.9726	**0.003287**
F9-NS	0.355	0	**0.001329**	0.9964	0.005271
F9-S	7.456	7.101	0	**0.002482**	0.9401
F10-NS	0.8521	0.4971	6.604	0	**0.01034**
F10-S	6.391	6.036	1.065	5.539	0

Q-values of paired Tukey tests are shown below the diagonal, and *P* values above the diagonal. Using Bonferroni correction, a significant *P* value is <0.005.

ROCK: the Rockefeller strain; F9-S and F10-S: the selected line at ninth and tenth generations, respectively; F9-NS and F10-NS: the non-selected line at ninth and tenth generations, respectively.

Finally, relative to the ROCK strain, the fitness, which was calculated from *R_0_* and *r*, was 0.79 and 0.82 for the selected line at F9 and F10 generations, respectively; meanwhile, 0.95 and 0.97 for the non-selected line at F9 and F10 generations, respectively (Table 4).

**Table 4:** Relative fitness and parameters used to calculate it. Fitness was calculated relative to the ROCK strain.

	net reproductive rate per generation: *R_0_*	intrinsic rate of increase in days: *r*	generation time in days: G	*r* derived from Euler-Lotka equation	relative fitness
ROCK	203.65	26.23	0.203	0.27	1.00
F9-NS	176.16	27.49	0.188	0.26	0.95
F10-NS	152.50	25.46	0.198	0.26	0.97
F9-S	57.66	23.27	0.175	0.21	0.79
F10-S	51.70	18.80	0.209	0.22	0.82

*l_x_*: age specific survival, *m_x_*: fecundity. ROCK: the Rockefeller strain, F9-S and F10-S: the selected line at ninth and tenth generations, and F9-NS and F10-NS: the non-selected line at ninth and tenth generations.

## Discussion

Variation in body size affects fitness as it has been suggested by the strong correlation to one or several of its components, like fecundity or survival age specific [32]–[37] Wing length, is often used in mosquitoes as an estimator of body size [32], [36], [38], but it cannot always be demonstrated [39], [40]. We used wing centroid-size (CS) as estimator of global size, instead of wing length, because we found a positive correlation with dry body weight and because CS detects size changes in all directions, not only along the largest axis of wing [8].

Furthermore, we observed that field isolate with highest lambda-cyhalothrin resistance contained the smallest insects and the susceptible reference strain, the biggest one (Fig. 3). But the correspondence between lambda-cyhalothrin resistance to wing size was not perfect (Fig. 2). We observed that insects similar in wing size were found in isolates separated by 520 km, which covers three branches of the Andes mountain range and at least two of the largest rivers of the country (Fig. S1). Those municipalities have different altitudes, climates and history of *Ae. aegypti* colonization [41], [42]. Moreover, here we studied adults which emerged in the laboratory from larvae and pupae collected in the field. Our method of collection controlled maternal effects, but water containers where larvae and pupae were collected could have different microclimates. Then, besides lambda-cyhalothrin resistance, other factors like density, nutrition, temperature, and relative humidity could affect the wing size [43], [44]; thus, unmeasured environmental variables in addition to their own genetic factors could also affect the lambda-cyhalothrin resistance to wing size relationship. Related to possible seasonal effects due to the fact that collections of isolate spanned over a 13-month period (Table 1), we discarded them because this Neotropical region does not have significant oscillations along the year (see World Meteorological Organization at http://www.wmo.int/, for climate parameters of Quibdó and Cúcuta).

CS of the selected and the non-selected lines seem hard to change, because they did not change during nine generations relative to their parents; but in the F20 generation, individuals were significantly bigger, with the non-selected line contained the biggest ones (Fig. 5). This result seems to follow the same pattern of those observed in field where most of the isolates containing the smaller individuals exhibited the higher RR_50_ (Fig. 3), and the biggest individuals belonged to the ROCK susceptible strain.

Smaller size associated with increased resistance could be a disadvantage, if smaller individuals have decreased fecundity because of reduced ability to ingest blood or having fewer ovarioles [36]. In *Culex pipiens*, the organophosphate resistance was associated with a smaller adult size [12], [45]; but *Ae. albopictus* resistance to permethrin was associated with larger size when compared with the susceptible strain [46], [47]. *Aedes aegypti* females exposed to sublethal doses of Spinosad were significantly larger than control females; male survivors, in contrast, were significantly smaller than controls [48]; although, resistance level to Spinosad was not reported. Thus, it seems that the family Culicidae has not a stable response in body size related to insecticide resistance.

The significant increase in wing size of both the selected and the non-selected lines at F20 generation relative to their parent population (Fig. 5) may reflect the ideal conditions of growth in the laboratory. However, Jirakanjanakit and Dujardin [49] observed a progressively significant reduction in size of four *Ae. aegypti* lines with the lifetime spent in laboratory from 17 to 564 generations. We found it difficult to explain these conflicting results, but perhaps the most likely explanation rests in genetic drift and inbreeding effects and/or different micro-environmental conditions of different laboratories.

As far as we know, a lot of attention has been paid to size of mosquitoes and its relationship with insecticide resistance and fitness, but nothing between their shape, resistance and fitness. However, shape has proven to be more stable, less labile than size and expresses more about genetics and evolution of organisms [9], [50]. In particular, wing shape of *Ae. aegypti* has shown more consistent evidence for genetic determinism than wing size [42], [51].

In this work, wing shape matched very well with geographic origins of isolates and with lambda-cyhalothrin resistance (Fig. 4). Although differentiation in wing shape across large distances occurs, it has been shown [42] that different populations of *Ae. aegypti* belong to the same species, and are distinct from the closely related species *Ae. albopictus*. Wing shape differentiation observed here could be related to the history of colonization of *Ae. aegypti* in Colombia. Before 1952, *Ae. aegypti* was widely distributed throughout the country and by the 1960 s it was eradicated completely from Colombia, except for the city of Cúcuta in the east, bordering Venezuela. Unfortunately, in 1971 there was a new and intensive re-infestation from the northern port cities of Colombia [41].

In addition to the association between wing shape and geography, we observe a second level of significant relationship between wing shape and resistance to lambda-cyhalothrin (Fig. 4). As far as we know, there are no current studies about mosquitoes' shape changes in response to insecticides resistance in these biological vectors; and in other insects there are few [52]. Our results also demonstrate the importance of focusing on shape more than in size when studying the morphological response to insecticide resistance in mosquitoes.

We observed in the F9 generation that the selected and the non-selected lines had a very different wing shape than that of their parents, but it was not different between the two lines (Fig. 6). However, in the F20 generation, the selected and the non-selected lines were significantly separated between them and with the F9 generation and its parents. We saw in this behavior the joint effects of laboratory colonization and the response to selection. We think that wing shape changes are easier when an organism suffers a bottleneck and founder effects (likely at initial conditions of laboratory colonization) and are more difficult in response to selection to insecticide-resistance.

Wing shape in each isolate was statistically different from others, with one exception (Table 2), but the wing size effects on wing shape (allometry) could not be discarded. Because a common allometric slope to all groups was not found, we could not verify whether wing shape variation was not simply the passive consequence of wing size variation [8]. We repeated the analysis excluding the ROCK strain because we think that the peculiar history of this reference strain could distort the model. But again, we could not find a common allometric slope. Therefore, we interpreted that isolates studied here did not share a common allometric growth. The different histories between Cúcuta and Quibdó isolates could also account their different allometries.

When we examined the allometries of the non-selected and the selected lines, we found that wing size significantly contributed to wing shape variation and that a common allometric slope could be found. Then, we could examine the variation in wing shape, scaling it by wing size, and we concluded that the lab's selection for resistance to lambda-cyhalothrin generated differences in wing shape that were not a passive consequence of variation in wing size.

Generally, insecticide-resistance is associated with negative effects on fitness [53], [54]. It is not easy to measure all components of fitness, but we measured probably the most important for quantifying the costs of lambda-cyhalothrin resistance in *Ae. aegypti*. The parental COM isolate showed an initial RR_50_ of 24.23 times relative to the susceptible ROCK strain, which was interpreted as a high level of natural resistance to this pyrethroid. Throughout the lab's selection we could raise this RR_50_ to 225.4X, 91.53X and 114.25X for F9, F10 and F20 generations, respectively. However, the parallel line not subjected to selection showed a progressive decrease in resistance until 1.95X. Such a change from a moderate resistance to susceptibility suggests that resistance to lambda-cyhalothrin carries a cost in absence of the insecticide.

We observed different levels of resistance to lambda-cyhalothrin in field isolate from the two municipalities evaluated (Table 1). The schemes of the use of insecticides in Cúcuta and Quibdó are different. Cúcuta employs mainly lambda-cyhalothrin, periodically spraying throughout all the year and covering the entire city. Quibdó employs mainly malathion, an organophosphate, which is only used when and where cases are detected. In this municipality, lambda-cyhalothrin is also used, but with less frequency than malathion. On the other hand, previous work showed that the selected line had cross-resistance to other pyrethroid (permethrin) but not to the organophosphates temephos and malathion [55], a situation which suggests that rotation of both families of insecticides is an appropriate alternative for managing the insecticide-resistance evolution.

Even the proximate collections within each municipality exhibited distinct resistance levels (Table 1). The scheme of collections could help to understand this phenomena. Given that the flight range of *Ae. eagypti* has been calculated in 840 m [56], we collected the insects in isolates separated by 1.5 to 5 km. Then each isolate could have been experienced genetic drift when they were founded by few adult survivors of lambda-cyhalothrin control. On the other hand, microarray analyses showed that laboratory adaptation to the pyrethroid permethrin in *Ae. aegypti* is genetically complex, largely conditioned by pre-existing target site insensitivity in the *para* gene and dependent on the geographic origin of the isolates [57]. Maybe, lab adaptation to the pyrethroid lambda-cyhalothrin behaves similarly. Furthermore, differential pressures from non-planned use of pyrethroids at domestic level, the non-standardized use of lambda-cyaholothrin impregnated bednets, and indirect exposure to xenobiotics could also influence such different resistance levels [20].

Related to the dramatic decrease in RR_50_ of the selected line from F9 to F10, and then increased in F20 to nearly half of the F9, Saavedra-Rodríguez et al [57] found a similar trend. They selected six field isolates of *Ae. aegypti* with permethrin for five generations in laboratory and registered a progressive increase in resistance levels except for two isolates, in which the resistance levels suddenly decrease in the fourth generation, and then raised again in the fifth generation. The authors observed an important amount of uniquely transcribed genes in individual isolates and thought that the differential response to selection of insecticide resistance could be associated with lethal or deleterious recessive alleles or even with genes related to metabolic resistance.

On the other hand, we do not discard a human error in the experimental protocols, but in spite the notorious oscillation, the selected F10 showed a RR_50_ of nearly nine times higher than the non-selected line, and the selected F20 of nearly 60 times. It is important remember that the selected and the non-selected lines were derived from the same parental insects, and that demographic parameters were studied in parallel on the F9 generation when RR_50_ reached the peak. The F10 and F20 generations were used to verify that under the selective regime, resistance was kept significantly different from the non-selected relatives, and to assess the corresponding morphometric changes.

To examine more deeply the fitness costs, we measured six recognized components of fitness, but only age-specific survival of females and fecundity showed significant differences between the selected and the non-selected lines (Table S3). We measured the components of fitness under ideal laboratory's conditions, which, although it did not reflect entirely the isolate dynamics on the field, is a way to study the biological potential of this species. Comparisons between non-related strains are one of the more common weaknesses in studies about fitness costs of resistant mosquitoes [12]; however, our work studied two lines of mosquitoes selected and non-selected for insecticide-resistance, which shared the same genetic background, which is fundamental to understand the evolutionary capabilities of *Ae. aegypti* and then planning optimal policies of vector control [7].

The survival probability of females, which were selected for resistance to lambda-cyhalothrin, quickly decreased in youngest individuals (10–30 days old); but afterwards, it progressively slowed its decline and some individuals could reach older ages (Fig. 7). The susceptible ROCK strain and the non-selected females displayed similar curves; meanwhile, the fecundity was significantly lower for the selected line (Fig. 8). It is important to notice that the ROCK strain and the non-selected line did not share a genetic background, and the analysis of life history components were performed at the same time and in the same environment. Moreover, both the selected and non-selected lines shared the same genetic background. Because of this, we think there were no other intrinsic triggers of mortality and fecundity between the selected and the non-selected lines than insecticide-resistance, and consequently it suggests costs in age-specific survival of females, and fecundity for *Ae. aegypti* resistant to lambda-cyhalothrin.

Fitness costs due to lower survival and fecundity in *Ae. aegypti* females associated with insecticide-resistance has been reported in several works [58]–[62], but this is the first time that two estimators of the per capita rate of increase are calculated (*R_0_* and *r*) and from them the relative fitness.

The statistical analyses of *R_0_* (net reproductive rate per generation) and *r* (intrinsic rate of increase in days) showed significant differences between the selected and the non-selected lines; besides, the *r* parameter did not show differences with the ROCK strain (Table 3). Setting a relative fitness value of 1.0 to the ROCK strain, the selected line reduced its fitness by 21%, meanwhile the non-selected line by only 4% (Table 4).

In conclusion, we were able to increase 10-fold the RR_50_ to lambda-cyhalothrin of a mosquito-line from COM isolate and held it in laboratory conditions, at least for two years. Furthermore, we observed that the parallel line, related to the selected one, and not subjected to insecticide pressure, reverted to its susceptibility status in just 20 generations. This information tells us about the evolutionary capacities of *Ae. aegypti* and strongly suggests that there are costs in fitness in absence of insecticides. Beserra et al. [63], [64] estimated in average between 21 to 31 the number of generations per year under natural conditions for several populations in Brazil. Thus in absence of lambda-cyhalothrin, reversion to susceptibility in the field could occur in less of one year, if we take in account that natural conditions are probably not as optimal as laboratory conditions. As a practical consequence the knowledge of reversion to insecticide-susceptibility in relative short time is useful to preserve the utility of the few current insecticides molecules currently available for vector control of tropical diseases.

Moreover, the present study showed the fitness disadvantage focused in less fecundity and survival, but not in the average hatchability, average time to pupation, average time to eclosion adults, and proportion of males and females emerging. Such fitness costs could negatively affect the extrinsic incubation period of the dengue virus, altering the dynamic of the virus transmission by mosquitoes [65].

Wing shape is a more reliable marker of the resistance status of *Ae. aegypti* natural populations, at least for lambda-cyhalothrin, than wing size.

Mosquito control programs could benefit from the knowledge of natural resistance status of populations and from the fitness costs. If we know fitness costs of resistance to insecticides and time to revert to susceptibility in absence of insecticides, it might be possible to implement efficient strategies to combine insecticides over time and/or space to delay or prevent the evolution of resistance and supplement this with biological control and education of communities [6].

## Supporting Information

Figure S1 **Map of Colombia showing the locations of municipalities of Cúcuta and Quibdó and the locations of isolates within both municipalities.** The map of Colombia was downloaded from the public domain at the CIA (https://www.cia.gov/library/publications/the-world-factbook/index.html). The maps of Cúcuta and Quibdó were opened in the OpenStreetMap's viewer and exported as PNG image files (http://www.openstreetmap.org/). The points and labels of locations were edited with the GIMP software ver. 2.8 (http://www.gimp.org/).
(EPS)

Figure S2 **Scatter plot of the scores along the first two principal components (relative warps), the convex hull for each group and the average scores for each group.** Convex hulls enclose individuals from nine natural isolates which were ordered on the two first relative warps. To easy viewing of the group's centroids, the individual positions are not shown. Deformation grids for the extreme values of the first and the second relative warps on the mean configuration, are also shown. The isolates of the municipality of Cúcuta are represented by red tones and the isolates of the municipality of Quibdó are represented by green tones.
(EPS)

Table S1 **Changes in susceptibility to lambda-cyhalothrin of the selected line relative to the susceptible reference ROCK strain.**
(DOC)

Table S2 **Changes in susceptibility to lambda-cyhalothrin of the non-selected line relative to the susceptible reference ROCK strain.**
(DOC)

Table S3 **Summary of parameters which did not show statistical differences (** ***P*** **>0.05) among the reference susceptible ROCK strain, and the selected and the non-selected lines at F9 and F10 generations.** ROCK: reference susceptible ROCK strain; F9-S and F10-S: the selected line at F9 and F10 generations, respectively; F9-NS and F10-NS: the non-selected line at F9 and F10 generations, respectively. Between parenthesis: standard deviation.
(DOC)
