# Supplementary material for: Geometric Morphometrics of Nine Field Isolates of Aedes aegypti with Different Resistance Levels to Lambda-Cyhalothrin and Relative Fitness of One Artificially Selected for Resistance
Source: PLoS One. 2014 May 6;9(5):e96379. doi: 10.1371/journal.pone.0096379 (PMC4011790; doi:10.1371/journal.pone.0096379)
Supplement: Table S1 — Changes in susceptibility to lambda-cyhalothrin of the selected line relative to the susceptible reference ROCK strain. (DOC) [file pone.0096379.s003.doc]

TABLE S1. Changes in susceptibility to lambda-cyhalothrin of the selected line relative to the susceptible reference ROCK strain.

| **gen** | **LC_50_** | **CI95%** | **RR_50_** | **LC_90_** | **CI95%** | **RR_90_** | **slope (sd)** | **Χ^2^ (df)** | ***P*** | **Regression equation** | **Mortality adults (%)** |
| --- | --- | --- | --- | --- | --- | --- | --- | --- | --- | --- | --- |
| ROCK | 0.0004 | 0.00033 - 0.00046 | 1 | 0.00134 | 0.00113 - 0.00169 | 1 | 2.43 (0.23) | 1.59 (3) | 0.33 | y = 5.51 + 2.43(x – 6.81) | 100 |
| F1 | 0.00969 | 0.00923 - 0.01010 | 24.22 | 0.01568 | 0.01448 - 0.01742 | 11.70 | 6.13 (0.49) | 7.75 (3) | 0.95 | y = 5.01 + 6.13(x – 7.99) | 100 |
| F2 | 0.00989 | 0.00927 - 0.01054 | 24.72 | 0.02139 | 0.01909 - 0.02476 | 15.96 | 3.82 (0.28) | 5.44 (4) | 0.76 | y = 5.021 + 3.82(x – 7.99) | 99 |
| F3 | 0.00978 | 0.00926 - 0.01033 | 24.45 | 0.01702 | 0.01558 - 0.01912 | 12.70 | 5.32 (0.43) | 1.82 (3) | 0.39 | y = 5.03 + 5.32(x – 7.99) | 100 |
| F4 | 0.013 | 0.01215 - 0.01392 | 32.5 | 0.02308 | 0.02078 - 0.02649 | 17.22 | 5.14 (0.42) | 0.32 (2) | 0.15 | y = 4.93 + 5.14(x – 8.10) | 92 |
| F5 | 0.01771 | 0.01638 - 0.01899 | 44.27 | 0.02666 | 0.02451 - 0.02981 | 19.89 | 7.21 (0.75) | 4.81 (4) | 0.81 | y = 5.29 + 7.21(x – 7.99) | 58 |
| F6 | 0.01617 | 0.01461 - 0.01786 | 40.42 | 0.03121 | 0.02729 - 0.03733 | 23.29 | 4.49 (0.42) | 0.65 (2) | 0.65 | y = 5.29 + 4.49(x – 8.22) | 42 |
| F7 | 0.02461 | 0.02199 - 0.02723 | 61.52 | 0.0681 | 0.05791 - 0.08394 | 50.82 | 2.88 (0.22) | 0.88 (2) | 0.88 | y = 5.07 + 2.88(x – 8.41) | 46 |
| F8 | 0.06299 | 0.05511 - 0.07462 | 157.4 | 0.16415 | 0.12481 - 0.25643 | 122.5 | 3.08 (0.40) | 0.93 (3) | 0.93 | y = 4.66 + 3.08(x – 8.69) | 32 |
| F9 | 0.09016 | 0.08008 - 0.10302 | 225.4 | 0.28036 | 0.22295 - 0.38329 | 209.22 | 2.6 (0.22) | 0.51 (2) | 0.51 | y = 4.62 + 2.60(x – 8.84) | 25 |
| F-10 | 0.03661 | 0.03120 - 0.04285 | 91.52 | 0.11725 | 0.09308 - 0.16050 | 87.49 | 2.54 (0.22) | 2.04 (3) | 0.50 | y = 5.03 + 2.53(x – 5.77) | 32 |
| F-11 | 0.04059 | 0.03629 - 0.04540 | 101.48 | 0.09389 | 0.08039 - 0.11458 | 70.07 | 3.52 (0.29) | 7.72 (3) | 0.95 | y = 4.99 + 3.12(x – 8.61 | ND |
| F-12 | 0.03883 | 0.3484 - 0.04327 | 47.08 | 0.08539 | 0.07361 - 0.10533 | 63.18 | 3.74 (0.31) | 5.07 (3) | 0.83 | y = 5.00 + 3.75(x – 8.59) | ND |
| F-13 | 0.13188 | 0.11104 - 0.15979 | 329.7 | 0.48232 | 0.35589 - 0.74802 | 359.94 | 2.28 (0.24) | 6.42 (3) | 0.91 | y = 4.78 + 2.28(x – 9.02) | 15 |
| F-14 | 0.16367 | 0.13997 - 0.19611 | 409.2 | 0.05315 | 0.44899 - 0.91374 | 487.43 | 2.26 (0.22) | 2.19 (3) | 0.47 | y = 4.67 + 2.26(x – 9.07) | 29 |
| F-17 | 0.02548 | 0.02247 - 0.02880 | 64.2 | 0.06896 | 0.05800 - 0.08615 | 51.46 | 2.96 (0.24) | 5.29 ( | 0.84 | y = 5.07 + 2.96(x – 8.43) | 32 |
| F-18 | 0.09078 | 0.07317 - 0.11279 | 226.95 | 0.42388 | 0.31381 - 0.62657 | 316.33 | 1.92 (0.16) | 0.51 (2) | 0.22 | y = 4.98 + 1.92(x – 8.95) | 15 |
| F-19 | 0.03163 | 0.02752 - 0.03616 | 79.08 | 0.07505 | 0.06281 - 0.09525 | 50.01 | 3.42 (0.33) | 1.59 (3) | 0.34 | y = 5.10 + 3.42(x – 8.53) | 37 |
| F-20 | 0.04569 | 0.03899 - 0.05395 | 114.23 | 0.15475 | 0.12077 - 0.21575 | 115.49 | 2.42 (0.22) | 1.53 (4) | 0.18 | y= 4.88 + 2.42(x – 8.61) | 8 |
