# Supplementary material for: Geometric Morphometrics of Nine Field Isolates of Aedes aegypti with Different Resistance Levels to Lambda-Cyhalothrin and Relative Fitness of One Artificially Selected for Resistance
Source: PLoS One. 2014 May 6;9(5):e96379. doi: 10.1371/journal.pone.0096379 (PMC4011790; doi:10.1371/journal.pone.0096379)
Supplement: Table S2 — Changes in susceptibility to lambda-cyhalothrin of the non-selected line relative to the susceptible reference ROCK strain. (DOC) [file pone.0096379.s004.doc]

TABLE S2. Changes in susceptibility to lambda-cyhalothrin of the non-selected line relative to the susceptible reference ROCK strain.

| **gen** | **LC_50_** | **CI95%** | **RR_50_** | **LC_90_** | **CI95%** | **RR_90_** | **slope (sd)** | **Χ^2^*(df)*** | ***P*** | **Regression equation** | **Mortality adults (%)** |
| --- | --- | --- | --- | --- | --- | --- | --- | --- | --- | --- | --- |
| ROCK | 0.0004 | 0.00033 - 0.00046 | 1 | 0.00134 | 0.00113 - 0.00169 | 1 | 2.43 (0.23) | 1.59 | 0.33 | y = 5.51 + 2.43(x – 6.81) | 100 |
| F1 | 0.00969 | 0.00923 - 0.01018 | 24.23 | 0.01568 | 0.01448 - 0.01742 | 11.701 | 6.13 (0.49) | 7.75 | 0.95 | y = 5.00 + 6.13(x – 7.98) | 100 |
| F2 | 0.00781 | 0.00733 - 0.00831 | 19.53 | 0.01461 | 0.01335 - 0.01636 | 10.9 | 4.71 (0.34) | 7.53 | 0.94 | y = 5.14 + 4.71(x – 7.99) | 96.3 |
| F3 | 0.00629 | 0.00577 - 0.00679 | 15.73 | 0.01292 | 0.01169 - 0.01474 | 9.64 | 4.09 0.31) | 0.44 | 0.06 | y = 5.32 + 4.09(x – 7.87) | 99 |
| F4 | 0.0048 | 0.00425 - 0.00532 | 12.00 | 0.01052 | 0.00938 - 0.01219 | 7.85 | 3.76 (0.34) | 4.3 | 0.78 | y = 5.49 + 3.76(x – 7.81) | 98 |
| F5 | 0.00357 | 0.00294 - 0.00409 | 8.93 | 0.00808 | 0.00704 - 0.00969 | 6.03 | 3.61 (0.43) | 1.63 | 0.34 | y = 5.69 + 3.61(x – 7.74) | 100 |
| F6 | 0.00501 | 0.00435 - 0.00583 | 12.53 | 0.01345 | 0.01079 - 0.01822 | 10.04 | 2.99 (0.29) | 2.30 | 0.49 | y = 4.87 + 2.99(x – 7.65) | 100 |
| F7 | 0.00355 | 0.00318 - 0.00396 | 8.88 | 0.00798 | 0.00685 - 0.00971 | 5.96 | 3.64 (0.3) | 3.90 | 0.72 | y = 4.98 + 3.64(x – 7.54) | 100 |
| F8 | 0.00492 | 0.00430 - 0.00569 | 12.30 | 0.01845 | 0.14470 - 0.02544 | 13.77 | 2.23 (0.18) | 1.46 | 0.31 | y = 4.81 + 2.23(x – 7.61) | 99.5 |
| F9 | 0.004 | 0.00353 - 0.00449 | 10.00 | 0.01164 | 0.01004 - 0.01395 | 8.69 | 2.76 (0.19) | 2.40 | 0.51 | y = 5.28 + 2.76(x – 7.70) | 99.5 |
| F-10 | 0.00432 | 0.00409 - 0.00455 | 10.80 | 0.00543 | 0.00507 - 0.00605 | 4.052 | 3.83 (0.32) | 2.37 (3) | 0.50 | y = 4.98 + 3.83(x – 7.68) | 100 |
| F-11 | 0.00718 | 0.00634 - 0.00803 | 17.95 | 0.01775 | 0.01549 - 0.02097 | 13.25 | 3.30 (0.25) | 3.45 (1) | 0.94 | y = 5.34 + 3.26(x – 7.68) | 100 |
| F-13 | 0.00458 | 0.00404 - 0.00520 | 11.45 | 0.01007 | 0.00850 - 0.01265 | 7.52 | 3.75 (0.36) | 1.25 (3) | 0.26 | y = 4.99 + 3.75(x – 7.66) | 100 |
| F-18 | 0.00412 | 0.00380 - 0.00440 | 10.3 | 0.00657 | 0.00614 - 0.00715 | 4.90 | 6.34 (0.59) | 1.63 (3) | 0.35 | y = 5.60 + 6.34(x – 7.71) | 70 |
| F-20 | 0.00078 | 0.00066 - 0.00090 | 1.95 | 0.00188 | 0.00157 - 0.00244 | 1.40 | 3.34 (0.39) | 5.22 (3) | 0.84 | y = 5.30 + 3.34(x – 6.98) | 98 |
