# Supplementary material for: Geometric Morphometrics of Nine Field Isolates of Aedes aegypti with Different Resistance Levels to Lambda-Cyhalothrin and Relative Fitness of One Artificially Selected for Resistance
Source: PLoS One. 2014 May 6;9(5):e96379. doi: 10.1371/journal.pone.0096379 (PMC4011790; doi:10.1371/journal.pone.0096379)
Supplement: Table S3 — Summary of parameters which did not show statistical differences ( P >0.05) among the reference susceptible ROCK strain, and the selected and the non-selected lines at F9 and F10 generations. ROCK: reference susceptible ROCK strain; F9-S and F10-S: the selected line at F9 and F10 generations, respectively; F9-NS and F10-NS: the non-selected line at F9 and F10 generations, respectively. Between parenthesis: standard deviation. (DOC) [file pone.0096379.s005.doc]

Table S3. Summary of parameters which did not show statistical differences (*P* > 0.05) among the reference susceptible ROCK strain, and the selected and the non-selected lines at F9 and F10 generations. ROCK: reference susceptible ROCK strain; F9-S and F10-S: the selected line at F9 and F10 generations, respectively; F9-NS and F10-NS: the non-selected line at F9 and F10 generations, respectively. Between parenthesis: standard deviation.

| PARAMETER |  | ROCK | F9-NS | F10-NS | F9-S | F10-S |
| --- | --- | --- | --- | --- | --- | --- |
| Average time to develop pupae (days) |  | 6.04 (0.45) | 5.21 (0.09) | 5.53 (0.32) | 5.47 (0.11) | 6.17 (0.19) |
| Average time to develop adults (days) | | Males | | --- | | Females | | | 7.92 (0.17) | | --- | | 8.32 (0.20) | | | 7.11 (0.15) | | --- | | 7.11 (0.05) | | | 7.10 (0.06) | | --- | | 7.47 (0.44) | | | 7.07 (0.14) | | --- | | 7.16 (0.12) | | | 7.47 (0.24) | | --- | | 7.54 (0.35) | |
| Ratio of males on females emergence |  | 0.97 (0.37) | 1.10 (0.21) | 1.06 (0.5) | 0.83 (0.17) | 0.75 (0.36) |
| Hatchability |  | 0.80 (0.04) | 0.80 (0.04) | ND | 0.79 (0.05) | ND |
